# Hepatoprotective effect of a novel lactic acid‐fermented garlic extract functional food product against acute liver injury

**DOI:** 10.1002/fsn3.1385

**Published:** 2020-01-05

**Authors:** Tae Min Kim, Ki Hoon Kim, Jeong Hyun Jo, Joonghoon Park, Yong Sam Kwon, Je Hoon Yang

**Affiliations:** ^1^ Graduate School of International Agricultural Technology Seoul National University Pyeongchang Korea; ^2^ Institutes of Green‐Bio Science and Technology Seoul National University Pyeongchang Korea; ^3^ Research Center Dong‐A Pharmaceutical Co., Ltd. Yongin Korea; ^4^ Laboratory Animal Research Center Samsung Medical Center Seoul Korea

**Keywords:** acute liver injury, hepatoprotective functional food

## Abstract

Lactic acid‐fermented garlic extract (LAFGE) has been shown to have hepatoprotective role in liver diseases. This study was conducted to evaluate the efficacy of a new LAFGE‐based hepatoprotective functional food product (named D‐18‐007) formulated with other additive components, including l‐arginine, l‐ornithine, and the leaf extract of licorice and artichoke. In a rat model of d‐galactosamine(GalN)/LPS‐induced liver injury, the survival was significantly higher in animals treated with D‐18‐007 than in animals treated with LAFGE. The hepatic injury was alleviated by either LAFGE or D‐18‐007, but the overall effect was more significant in D‐18‐007, as shown by the necrosis, histology, and serum analyses. Also, the decrease in GalN/LPS‐induced lipid peroxidation in the liver tissue was more significant in D‐18‐007 than LAFGE. The decrease in IL‐6 protein in the liver was similar between LAFGE and D‐18‐007. Moreover, we compared the amount of the bile in normal animals and found that D‐18‐007 has better choleretic activity than LAFGE. Using this acute liver injury model, our results suggest that D‐18‐007 has an enhanced hepatoprotective effect in acute liver injury compared with LAFGE alone.

## Graphical abstract

In this study, we evaluated the efficacy of a lactic acid‐fermented garlic extract‐based hepatoprotective functional food formulated with l‐arginine, l‐ornithine, and the leaf extract of licorice and artichoke. In a rat model of acute liver injury, an enhanced hepatoprotective effect was found compared with garlic extract alone.

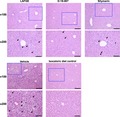

## 1 INTRODUCTION

Fulminant hepatic failure (FHF) is a life‐threatening condition with high mortality (80%–90%) that is caused by a sudden decrease in liver function resulting from the mass death of hepatocytes (Halegoua‐De Marzio & Sass, 2017). Fulminant hepatic failure can be induced by multiple factors, such as hepatotoxic drugs, alcohol, viral hepatitis, and bacteria, all of which can lead to various pathological conditions, including coagulopathy, encephalopathy, and multiple organ failure (Forbes & Newsome, 2016). Although the prognosis of FHF has been dramatically improved by liver transplantation, the development of a cost‐effective method for hepatic regeneration has been lacking (O'Grady, 2014). As a FHF model, rodent model of acute liver injury induced by d‐galactosamine (d‐GalN) and lipopolysaccharide (LPS) is often used (Shin et al., 2011). d‐GalN inhibits the production of uridine triphosphate, resulting in hepatic apoptosis via metabolic dysfunction and lipid peroxidation; LPS triggers proinflammatory cytokine production in inflammatory and Kupffer cells, causing acute liver injury.

So far, various in vitro and in vivo experiments have shown that natural plant products such as flavonoids, oils, alkaloids, and carotenoids can be effectively used to alleviate hepatotoxicity. Garlic (*Allium sativum*) is a phytomedicine that has long been used for therapeutic purposes throughout human history (Sendl, 1995). Garlic contains oil‐soluble organosulfur compounds (OSCs) such as diallyl sulfide, as well as water‐soluble OSCs like cycloalliin and S‐allyl cysteine (SAC). It has been previously reported that the hepatoprotective function of garlic can be attributed to garlic‐derived OSCs (Guan, Zhao, Xie, & Zeng, 2018). These OSCs have been reported to increase antioxidants such as glutathione, catalase, and GSH peroxidase in HFD‐induced obese mice (Lin & Yin, 2008). Various garlic preparations, including crude garlic (Abdel‐Salam & Sayed, 2012), garlic oil (El‐Khayat et al., 2010), and lactic acid bacteria‐fermented garlic extracts (LAFGE) (Choi et al., 2014; Jung et al., 2013), have been shown to promote hepatoprotection. Depending on the lactic acid bacteria, LAFGE has been reported to contain varying amount of OSCs including alliin, cycloalliin, S‐ethyl cysteine (SEC), SAC, and S‐methylcysteine (SMC) (Jung et al., 2013). Indeed, the hepatoprotective effect of LAFGE has been well‐reported in several liver disease models (Guan et al., 2018; Lee et al., 2016; Shin et al., 2014).

Artichoke (*Cynara scolymus*), a perennial plant originating from the Mediterranean, has long been used in traditional medicine to treat hepatobiliary diseases (Lattanzio, Kroon, Linsalata, & Cardinali, 2009). A modern therapeutic use of this herbaceous plant has been recognized due to its choleretic effect (Lattanzio et al., 2009; Saenz Rodriguez, Garcia Gimenez, & de la Puerta Vazquez, 2002). Additionally, recent studies have shown that the artichoke also has hepatoprotective, antimicrobial, and antioxidative effects in various in vitro and animal studies (El Morsy & Kamel, 2015; Gebhardt, 1997; Vamanu, Vamanu, Nita, & Colceriu, 2011). Licorice (*Glycyrrhiza glabra*) has been used in the past as a soothing herb in traditional medicine, but nowadays it has a wide range of applications, including as a sweetener in foods, in tobacco manufacturing, and also as a functional health food. Most importantly, it is well‐known for its hepatoprotective, antioxidative, antimicrobial, anti‐inflammatory, and immune‐regulatory activities (Kao, Wu, & Yen, 2014; Wang, Yang, Yuan, Liu, & Liu, 2015; Yang, Yuan, Ma, Zhou, & Liu, 2017). l‐arginine is the precursor of nitric oxide (NO) and has been known to play various roles in animals including hepatoprotection (Kurokawa et al., 2012; Ozsoy et al., 2011; Stuehr, 2004); l‐ornithine is known for its role in ammonia removal and thus is currently used to treat cirrhosis in humans (Butterworth & Canbay, 2019; Durgun et al., 2015). We have recently developed an advanced hepatoprotective food product named D‐18‐007, a LAFGE (Choi et al., 2014)‐based product that had been reformulated to contain the extracts of artichoke and licorice as well as l‐arginine and l‐ornithine. The extract of artichoke and licorice has also been registered as food ingredient by Korea Food and Drug Administration (KFDA), which led us to develop this new product without concerning safety issue. Herein, we evaluated whether D‐18‐007 has an enhanced hepatoprotective effect over LAFGE alone in a rat model of acute liver injury induced by d‐GalN/LPS.

## 2 MATERIALS AND METHODS

### 2.1 Reagents

LPS (*Escherichia coli* 0111:B4) and Ursodeoxycholic acid (UDCA) were purchased from Sigma‐Aldrich Inc. d‐galactosamine was purchased from Tokyo Chemical Industry Co., Ltd. Silymarin (Legalon^®^) was purchased from Bukwang Pharmaceuticals Inc. Silicone Solocath catheters (3Fr) were purchased from Harvard Apparatus Inc.

### 2.2 Preparation of D‐18‐**007**

D‐18‐007 was formulated with lactic acid‐fermented garlic extract, which is the main functional food ingredient, along with other ingredients including l‐arginine, l‐ornithine, artichoke extract, and licorice extract using a propriety method.

### 2.3 Animal experiments

All experiments were approved by the Institutional Animal Care and Use Committee (IACUC) of Seoul National University (reg. nos. SNU‐190702‐1, SNU‐180517‐2‐1). All procedures were conducted in accordance with the *Guide for the Care and Use of Laboratory Animals* (Institute for Laboratory Animal Research, 2011).

All animals were purchased from Koatech Inc. and housed at 23–24°C with a 12/12‐hr light/dark cycle. Male *SD* (Sprague Dawley) rats weighing 250–300 g were fasted for 16 hr and randomly assigned to four groups for the following intragastric administrations: (a) vehicle (3 ml), (b) LAFGE (150 mg/kg), (c) D‐18‐007 (2 ml/kg), and (d) Silymarin (100 mg/kg). After 1 hr of oral administration, acute liver injury was induced through intraperitoneal injection of GalN (900 mg/kg) and LPS (50 µg/kg). At 72 hr after GalN/LPS treatment, the animals were euthanized by being exposed to 4% isoflurane for 7–10 min. Blood was drawn from the right ventricle, and the liver was isolated for fixation with 10% NBF. Before conducting the efficacy test, we first assessed the survival rate for 72 hr after animals were treated to evaluate whether LAFGE or D‐18‐007 affected the survival of animals.

Bile flow was measured as described (Tonsberg et al., 2010). Briefly, rats were allocated into four groups and then treated the same way as described above, without inducing liver injury. After 1 hr, animals were anesthetized with 4% isoflurane/oxygen in a chamber. After the induction was confirmed, anesthesia was maintained with 2% isoflurane/oxygen via a nose cone. Animals then underwent laparotomy under a dissecting microscope (SMZ445, Olympus), and the skin was shaved. A hole was made in the proximal bile duct using a blade, and the beveled tip of a silicone SoloCath catheter (3 Fr) was inserted into the bile duct. To fix the catheter, a suture was made around the beads (6‐0 silk, Ethicon). The intestine was repositioned into the peritoneal cavity, and the peritoneal and muscle layers were closed with a continuous suture (Vicryl 5‐0, Ethicon) while ensuring that the free end of the catheter protruded out of the closure. Bile was steadily collected for 45 min into a 1.7 ml tube. Animals were then euthanized by CO_2_ asphyxiation.

### 2.4 Serum analyses

Whole blood was incubated at room temperature for 20 min. After centrifugation at 3,000 g for 20 min, serum samples were subjected to measurement of alanine aminotransferase (ALT) and aspartate aminotransferase (AST) enzymes using the Catalyst Dx Chemistry Analyzer system (IDEXX Veterinary Diagnostics). Rat TNF‐α and IL‐6 ELISA kits were purchased from BioLegend Inc., and the concentrations were measured as described in the manufacturer's instructions.

### 2.5 Measurement of lipid peroxidation and catalase activity in rat liver lysates

Measurements of thiobarbituric acid reactive substances (TBARS) and catalase were performed using liver homogenates from rats 72 hr after GalN/LPS injection. TBARS and Catalase kits were supplied by Cayman Chemical Company Inc. and BioVision Inc., respectively.

### 2.6 RNA extraction and reverse transcription‐quantitative polymerase chain reaction (RT‐qPCR) analysis

Total RNA was extracted from liver samples using Trizol^®^ (Thermo Fisher Scientific). cDNA was synthesized using a cDNA synthesis kit (PhileKorea) according to the manufacturer's instructions. Quantitative RT‐PCR was performed in tandem with the StepOnePlus™ Real‐Time PCR (Thermo Fisher Scientific). Changes in mRNA expression were determined according to the 2^−ΔΔCT^ method (Livak & Schmittgen, 2001). Primer sequences were as follows: MCP1, 5′‐CAGCCAGATGCAGTTAATGCC‐3′ and 5′‐AGCCGACTCATTGGGATCAT‐3′; IL‐1β, 5′‐CACCTCTCAAGCAGAGCACAG‐3′ and 5′‐GGGTTCCATGGTGAAGTCAAC‐3′; GAPDH, 5′‐TGGTGCTGAGTATGTCGTG‐3′ and 5′‐ AGTGATGGCATGGACTGTG‐3′.

### 2.7 Statistical analysis

All data were analyzed using GraphPad Prism 5.0 (GraphPad, San Diego, CA, USA). One‐way analysis of variance (ANOVA) followed by a post hoc Dunnett's test for multiple comparisons, and a Student's *t* test were used to compare the two groups. A *p*‐value of less than .05 was considered to be statistically significant.

## 3 RESULTS

### 3.1 D‐18‐007 is more effective in animal survival than LAFGE

**Figure 1:**
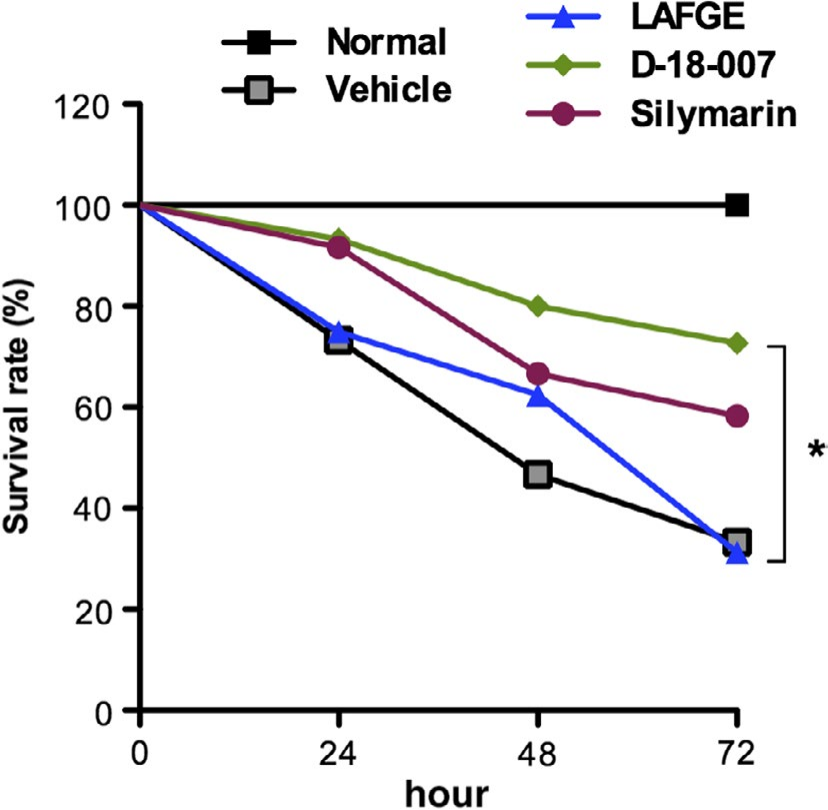
Effect of D‐18‐007 on the survival rate of GalN/LPS‐treated rats. Animals received an intraperitoneal injection of the vehicle, LAFGE, D‐18‐007, or silymarin 1 hr prior to GalN (900 mg/kg) and LPS (50 μg/kg) treatment. *p* < .05, *N* = 3 (normal) and 10 (other groups). *p* = .0294 and .0342 in 48 and 72 hr, respectively

To evaluate the hepatoprotective effect of D‐18‐007 against GalN/LPS‐induced mortality and hepatic injury in rats, we first investigated the survival rate for 72 hr after GalN/LPS administration. As shown in Figure 1, around 30% and 50% of animals died within 24 and 48 hr, respectively, in vehicle‐treated animals. In contrast, the mortality was significantly reduced in D‐18‐007‐treated animals against vehicle (*p* = .0294 and .0342 in 48 and 72 hr, respectively).

### 3.2 D‐18‐007 shows enhanced function in reducing liver injury markers than LAFGE

**Figure 2:**
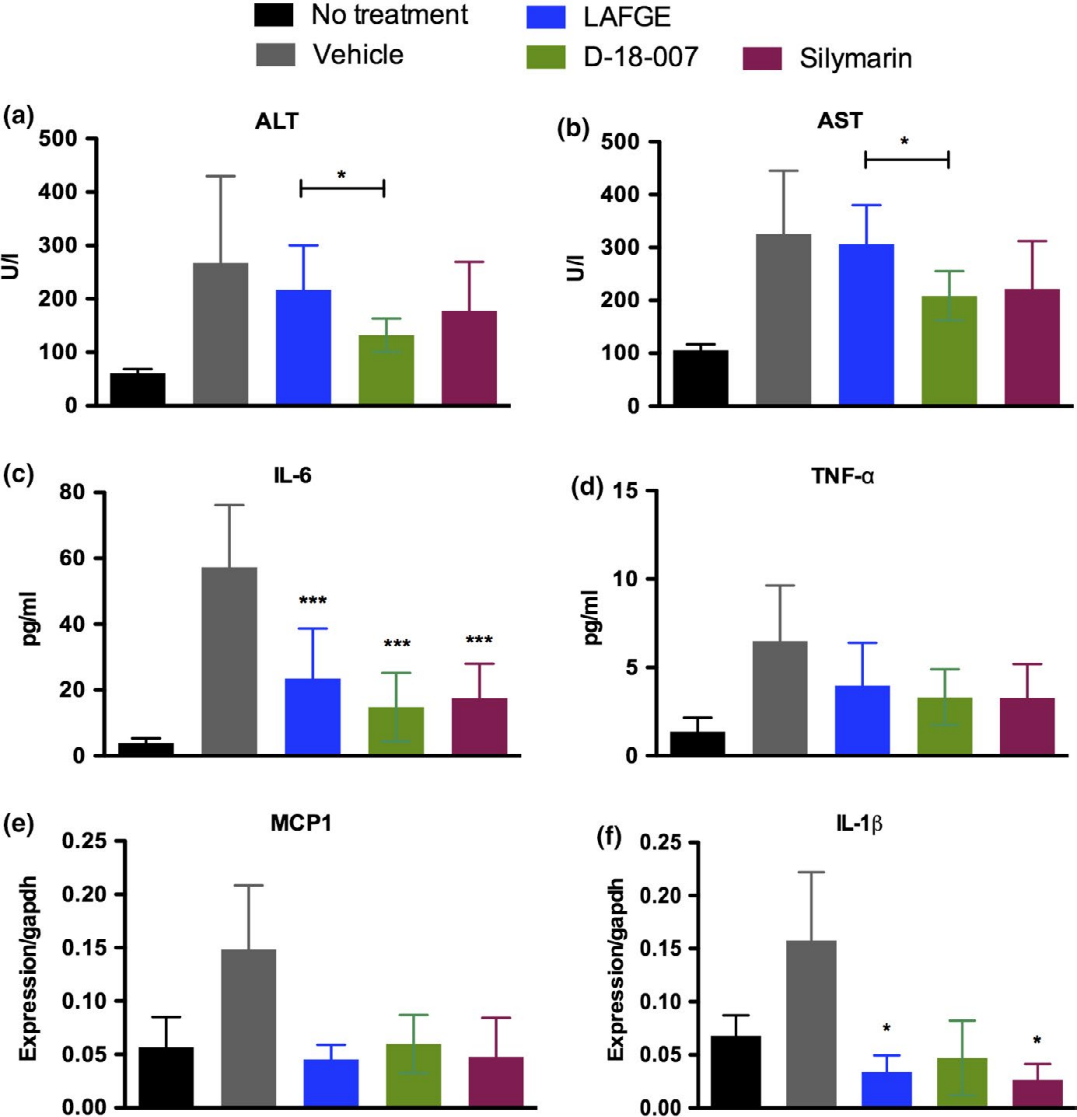
The effect of D‐18‐007 on alleviating liver inflammation induced by GalN/LPS. At 72 hr after GalN/LPS treatment, the serum levels of ALT and AST (a, b), along with IL‐6 and TNF‐α (c, d), were measured. The mRNA expression levels of MCP1 and IL‐1β (normalized against GAPDH) in the liver tissue were determined by qRT‐PCR (e, f). Data are mean ± *SD*. Student's *t* test was used to compare LAFGE‐ and D‐18‐007‐treated animals. *p < .05 and ***p < .005, *N* = 4 (no treatment) and 8 (other groups)

We next analyzed the serum levels of ALT and AST and found that GalN/LPS‐induced damage to the liver in vehicle‐treated rats. A slight reduction of both enzymes was noted in LAFGE‐treated animals; however, no significant change against vehicle was found (Figure 2a,b). In D‐18‐007‐treated rats, however, the serum levels of both enzymes were significantly decreased compared with those from LAFGE‐treated animals (ALT, *p* = .0221, LAFGE vs. D‐18‐007; AST, *p* = .0152, LAFGE vs. D‐18‐007). Also, a significant decrease of IL‐6 in the serum was found in LAFGE‐, D‐18‐007‐, and silymarin‐treated animals (Figure 2c, *p* = .009, .011, .021, respectively). No significant change was detected in TNF‐α among the groups (Figure 2d). The mRNA expression levels in the liver tissue were investigated using qRT‐PCR, and no statistical difference was found on the expression of MCP1 among animals treated with LAFGE, D‐18‐007, or silymarin (Figure 2e). The expression of IL‐1β was significantly lower in LAFGE‐ or silymarin‐treated animals compared with vehicle control (*p* = .0039 and .0051) (Figure 2f).

### 3.3 D‐18‐007 leads to an enhanced hepatic tissue recovery than LAFGE

**Figure 3:**
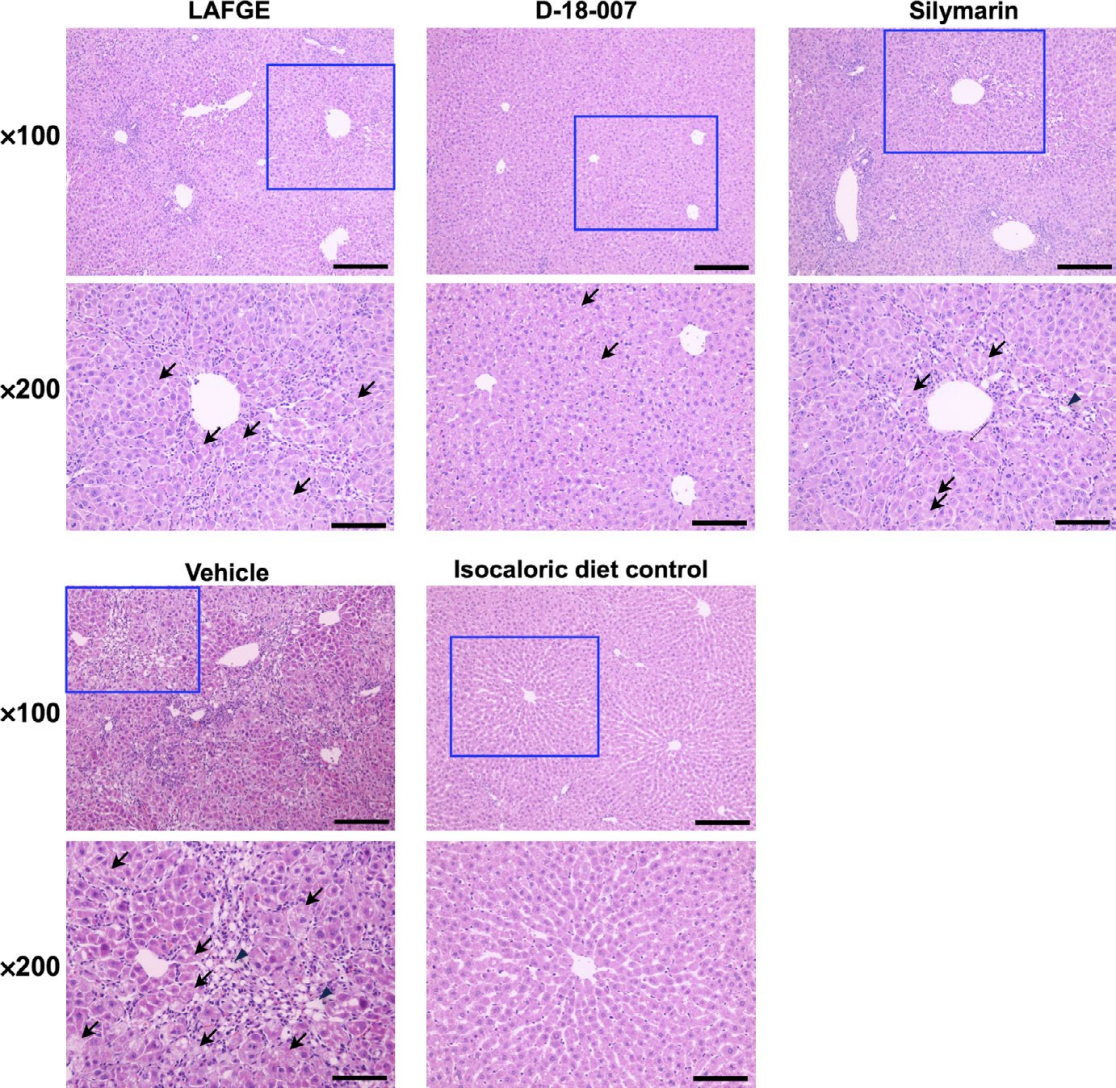
Representative H&E‐stained liver sections. Rats were pretreated with LAFGE, D‐18‐007, silymarin, or vehicle 1 hr prior to being administered GalN/LPS. At 72 hr, the left lobe was collected for histological analysis. Arrows and arrowheads indicate necrotic and ballooning hepatocytes, respectively. Scale bars are 50 and 25 µm in ×100 and ×200, respectively. *N* = 4 (no treatment) and 8 (other groups)

Histological analysis showed massive necrosis, a ballooning degeneration of hepatocytes, inflammatory cell infiltration, and lobular damage with loss of hepatic architecture 72 hr after GalN/LPS injection. Such pathology was attenuated in LAFGE‐treated animals, and even further ameliorated in the D‐18‐007‐treated group. In silymarin‐treated animals, the liver morphology was similar to those treated with LAFGE (Figure 3).

### 3.4 D‐18‐007 reduces lipid peroxidation and increases bile flow more potently than LAFGE

**Figure 4:**
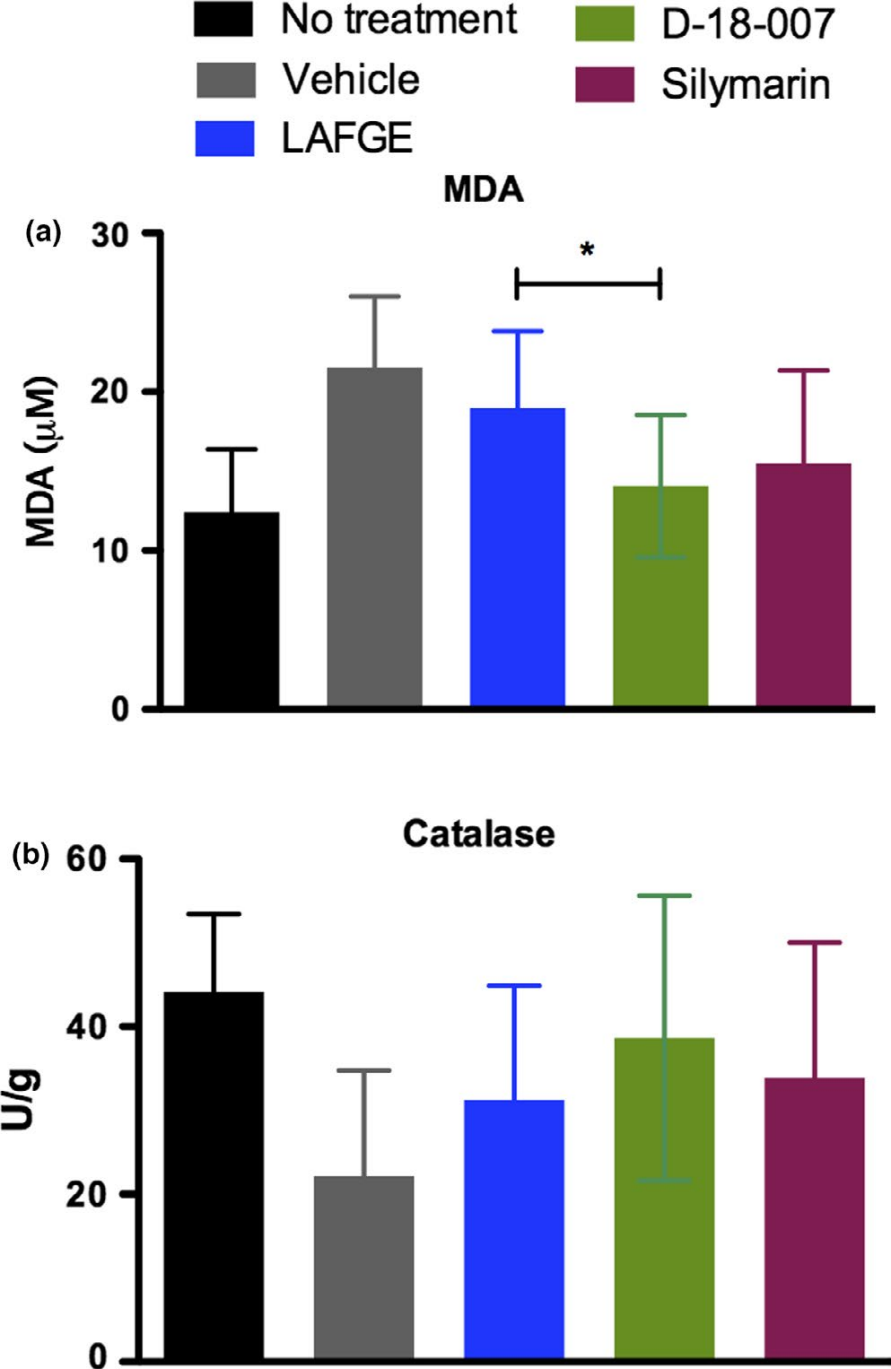
The effect of D‐18‐007 treatment on alleviating lipid peroxidation (a) and oxidative stress (b). TBARS and catalase activity in the liver tissue was measured 72 hr after GalN/LPS treatment. Student's *t* test was used to compare LAFGE‐ and D‐18‐007‐treated animals. *N* = 4 (no treatment) and 8 (other groups) for measuring TBARS and catalase activity. *N* = 10 per group for bile flow measurement. *p < .05. Data are mean ± *SD*. *N* = 10 per group

**Figure 5:**
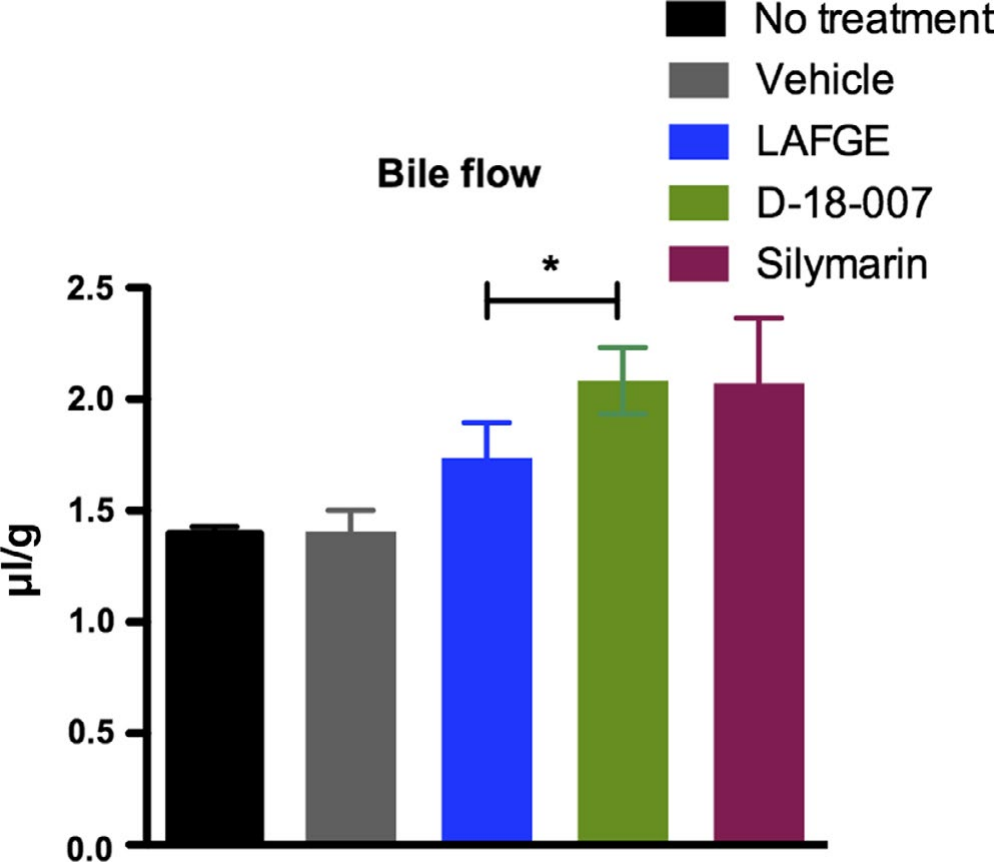
The effect of D‐18‐007 treatment on bile production. Student's *t* test was used to compare LAFGE‐ and D‐18‐007‐treated animals. *p = .0159. Data are mean ± *SD*. *N* = 10 per group

Figure 4a shows that GalN/LPS treatment increased lipid peroxidation, which was then reversed in animals treated with LAFGE and D‐18‐007. This reversal was more significant in D‐18‐007 compared with LAFGE (*p* = .0401). No significant change in the catalase activity in the liver tissue was found (Figure 4b). We next evaluated whether D‐18‐007 had a greater effect on the rate of bile production compared with LAFGE, since D‐18‐007 contains artichoke, which is known to increase bile secretion. As shown in Figure 5, animals treated with LAFGE, D‐18‐007, or silymarin exhibited enhanced bile flow during the study period. A significant increase in bile production was found in D‐18‐007‐treated animals compared with LAFGE‐treated animals (*p* = .0159).

## 4 DISCUSSION

So far, various acute hepatic injury models have been used to test the hepatoprotective role of garlic. For example, it was reported that pretreatment of aqueous garlic extract (AGE) was able to reduce liver injury and oxidative stress induced by GalN/LPS (El‐Beshbishy, 2008). Other study showed that the hepatic steatosis was blocked in rats treated with heat‐extracted aged black garlic in a high fat‐induced liver steatosis rat model (Shin et al., 2014). It was also reported that LAFGE attenuated acetaminophen‐induced liver injury by preventing apoptosis, inhibiting lipid peroxidation and increasing antioxidant enzymes (Lee et al., 2016). In the present study, we evaluated whether the hepatoprotective effect of LAFGE can be enhanced by adding l‐arginine, l‐ornithine, and the leaf extract of licorice and artichoke, using a rat model of GalN/LPS‐induced acute liver injury.

The hepatoprotective function of LAFGE has been reported due to its antioxidative properties, mainly attributed to the presence of organosulfur compounds like S‐allyl cysteine (SAC) and cycloalliin (Allison, Lowe, & Rahman, 2006). Cycloalliin in particular is resistant to heating, and relatively more stable during long‐term storage at room temperature compared with other organosulfur compounds found in garlic (Ichikawa et al., 2006). Our results showed that D‐18‐007 has higher potential to ease liver injury compared with LAFGE alone. Moreover, the bile flow rate was higher in animals treated with D‐18‐007 compared with those treated with LAFGE.

The beneficial effect of l‐arginine has been reported in several rodent models of hepatic injury. Using a hepatectomy model, Kurokawa et al. showed that l‐arginine treatment increased liver mass and the number of PCNA‐positive cells (Kurokawa et al., 2012). It was also shown that l‐arginine improved hepatic microcirculation following ischemia‐reperfusion injury (Uhlmann, Scommotau, Witzigmann, & Spiegel, 1998). Clinically, a mixture of l‐ornithine and l‐aspartate (LOLA) has been shown to have hepatoprotective properties in individuals with fatty liver diseases (Butterworth & Canbay, 2019). Such hepatoprotection seems to result from the transamination of l‐ornithine into glutamate (Butterworth & Canbay, 2019). Indeed, IV infusion of LOLA in patients with chronic liver disease induced an increase in plasma glutamate, and subsequently restored glutamine levels (Staedt et al., 1993). Thus, it is likely that the synthesis of glutamate from l‐ornithine in the liver may play a critical role in its hepatoprotective function for patients with chronic liver diseases. The product used in the present study contains the l‐arginine and l‐ornithine, and these two seem to have enhanced hepatoprotective function over LAFGE, although further analysis should be followed to clearly confirm its mechanism.

Since GalN/LPS is known to induce acute inflammation in the liver, we sought to examine the serum concentration of IL‐6 and TNF‐α. As results, IL‐6 was similarly decreased in animals treated with LAFGE, D‐18‐007 or silymarin, while no significant reduction in TNF‐α was found among all treated groups. Subsequent qRT‐PCR showed that the reduction of MCP1 mRNA expression was similar among animals treated with LAFGE, D‐18‐007, or silymarin compared with those treated with vehicle. Based on these results, it can be suggested that the anti‐inflammatory function does not differ between two functional foods. Another possibility would be the severity of the protocol that we have used for inducing liver injury, which may be too severe to reverse the disease progression during the study period. Therefore, further validation would be warranted to confirm whether D‐18‐007 has enhanced anti‐inflammatory function over LAFGE. Using a milder protocol by reducing the dosage of LPS would be an ideal option.

The increased bile flow in D‐18‐007‐treated animals may have been resulted from additional components other than LAFGE. Artichoke leaf extract has long been used for antidyspeptic activity, which is mediated by its well‐known choleretic activity (Ben Salem et al., 2015). It has been revealed that several components including flavonoid luteolin and its glycoside luteolin‐7O‐glucoside, and caffeoylquinic acids are the main players for its role (Adzet, Camarasa, & Laguna, 1987; Gebhardt, 1997). Specifically, it has been recognized that polyphenols within the extract may play key role in secreting cholephilic constituents into the bile (Gebhardt & Ueberham, 1998). Not only for these components, an active component of licorice liquiritigenin has been reported to have choleretic effect, and to induce transporters and hepatic phase‐II enzymes in LPS/GalN‐induced liver injury (Kim et al., 2009).

Our findings suggest that the hepatoprotective effect of D‐18‐007 is enhanced compared with LAFGE alone. Our preclinical data may contribute to developing LAFGE‐based hepatobiliary functional food. However, further studies may be required to identify the key differences in the underlying biological pathways, that is, those involved in tissue survival, apoptosis, or inflammation, between animals treated with D‐18‐007 and those treated with LAFGE. Additionally, it would be useful to test this product in other liver injury models, including high fat diet‐ or carbon tetrachloride (CCl_4_)‐induced hepatic injury, to determine whether its hepatoprotective effect is etiology‐specific.

## CONFLICT OF INTEREST

Authors declare no conflict of interest.

## ETHICAL APPROVAL

This study was approved by the Institutional Animal Care and Use Committee (IACUC) of Seoul National University.
